# DNA damage repair gene defects combined predict the efficacy of immune checkpoint inhibitors in metastatic castration-resistant prostate cancer: a retrospective cohort study

**DOI:** 10.3389/fgene.2026.1933643

**Published:** 2026-09-01

**Authors:** Kesong Qin, Gangqiang Hu, Erli Song

**Affiliations:** 1 Department of Urology, PKUCare CNOOC Hospital, Tianjin, China; 2 Department of Urology, Ezhou Central Hospital, Ezhou City, Hubei, China; 3 Department of Urology, Huai’an People’s Hospital of Hongze District, Huai’an City, Jiangsu, China

**Keywords:** biomarker, CDK12, DNA damage repair, homologous recombination repair, immune checkpoint inhibitors, metastatic castration-resistant prostate cancer, mismatch repair, PD-L1

## Abstract

**Background:**

Immune checkpoint inhibitors (ICIs) yield heterogeneous benefit in metastatic castration-resistant prostate cancer (mCRPC), underscoring the unmet need for robust predictive biomarkers. DNA damage repair (DDR) gene defects encompass mechanistically distinct subtypes—homologous recombination repair (HRR) deficiency, mismatch repair deficiency/microsatellite instability-high (MMRd/MSI-H), and CDK12 inactivation—that may differentially modulate anti-tumor immunity. This study evaluated the combined predictive value of DDR mutation profiles, tumor mutational burden (TMB), and PD-L1 expression for ICI efficacy in mCRPC.

**Methods:**

In this single-center retrospective cohort study conducted at PKUCare CNOOC Hospital (Tianjin, China), 152 patients with mCRPC who received ICI-containing therapy following ≥2 prior lines of systemic treatment were enrolled (January 2018–December 2023). Of these, 127 (83.6%) underwent tissue-based profiling with a validated 520-gene targeted NGS panel, and 25 (16.4%) were profiled by a validated 168-gene liquid biopsy panel. Patients were classified into four DDR subgroups: HRR-deficient, MMRd/MSI-H, CDK12-mutant, and DDR wild-type. Primary endpoints were objective response rate (ORR) and progression-free survival (PFS). Secondary endpoints included overall survival (OS), disease control rate (DCR), and prostate-specific antigen (PSA) response. Kaplan-Meier analysis, multivariate Cox regression, and ROC analysis were performed.

**Results:**

Among 152 patients, 101 (66.4%) harbored DDR alterations: 58 HRR-deficient (38.2%), 24 MMRd/MSI-H (15.8%), and 19 CDK12-mutant (12.5%). ORR was highest in MMRd/MSI-H (50.0%), followed by CDK12-mutant (26.3%), HRR-deficient (22.4%), and DDR wild-type (9.8%; p < 0.001). Median PFS was significantly longer across all DDR-mutant groups versus DDR wild-type (MMRd/MSI-H: 8.1 months; HR 0.31, 95% CI 0.18–0.53; p < 0.001). On multivariate analysis, DDR mutation subtype, TMB ≥10 mut/Mb, and liver metastasis were independent predictors of PFS. A composite biomarker integrating DDR status, TMB, and PD-L1 achieved the highest predictive accuracy (AUC 0.83, 95% CI 0.75–0.91). irAE rates did not differ significantly across subgroups (p = 0.614).

**Conclusion:**

DDR gene mutation profiling, integrated with TMB and PD-L1 expression, provides clinically actionable stratification of mCRPC patients for ICI therapy. A composite DDR/TMB/PD-L1 biomarker panel warrants prospective validation in dedicated clinical trials.

## 1 Introduction

Prostate cancer is the second most common malignancy and the fifth leading cause of cancer-related mortality in men worldwide. Metastatic castration-resistant prostate cancer (mCRPC) remains a lethal condition characterized by inevitable progression despite androgen deprivation therapy and next-generation hormonal agents. Although novel therapeutic strategies—including poly (ADP-ribose) polymerase (PARP) inhibitors and immune checkpoint inhibitors (ICIs)—have expanded the treatment landscape, durable responses are achieved in only a minority of patients, and predictive biomarkers remain inadequate (de Bono et al., 2020; Abida et al., 2020; Agarwal et al., 2023; Chi et al., 2023; Fizazi et al., 2023; Antonarakis et al., 2020).

ICIs targeting the programmed death-1/programmed death-ligand 1 (PD-1/PD-L1) axis and cytotoxic T-lymphocyte-associated protein 4 (CTLA-4) have transformed oncology across multiple tumor types; however, their efficacy in mCRPC has been largely disappointing except in molecularly defined subpopulations. The KEYNOTE-199 trial demonstrated an ORR of only 5% in unselected patients with mCRPC receiving pembrolizumab, highlighting the immunologically “cold” nature of most prostate tumors (Antonarakis et al., 2020; Kim and Koo, 2022; Lanka et al., 2023). Combination immunotherapy with nivolumab plus ipilimumab has also been investigated in mCRPC (Sharma et al., 2020). Conversely, patients with MSI-H or mismatch repair-deficient (MMRd) tumors derive substantial benefit from PD-1 blockade, underpinning the tissue-agnostic FDA approval of pembrolizumab for this molecularly defined subgroup (André et al., 2020; Marabelle et al., 2020).

DNA damage repair (DDR) encompasses a network of interrelated pathways—including homologous recombination repair (HRR), mismatch repair (MMR), nucleotide excision repair, and non-homologous end joining—that collectively maintain genomic integrity. Defects in these pathways are highly prevalent in mCRPC: somatic or germline alterations in BRCA1, BRCA2, ATM, and other HRR genes occur in approximately 20%–30% of patients, while CDK12 biallelic inactivation and MMR deficiency each occur in 5%–7% (Mateo et al., 2020; Annala et al., 2022). Given the clinical implications of inherited DDR alterations, consensus recommendations support germline testing for appropriately selected patients with prostate cancer (Giri et al., 2020). Beyond their established role as predictors of PARP inhibitor sensitivity, DDR defects create immunogenic tumor microenvironments through diverse mechanisms—including accumulation of cytosolic DNA, activation of the cGAS-STING innate immune pathway, increased neoantigen burden, and enhanced CD8^+^ T-cell infiltration (Peyraud and Italiano, 2020; Hanahan, 2022).

The three principal DDR subsets in mCRPC—HRR-deficient, CDK12-mutant, and MMRd/MSI-H—are mechanistically distinct and likely generate divergent immune phenotypes. MMRd/MSI-H tumors exhibit extreme hypermutation and the richest neoantigen landscape, explaining their exceptional responsiveness to PD-1 blockade. CDK12 biallelic loss drives tandem duplications, focal copy number gains, and intermediate TMB elevation with unique immune infiltration signatures (Antonarakis et al., 2020b; Reimers et al., 2020; Rescigno et al., 2021). HRR deficiency (particularly BRCA2 loss) impairs homologous recombination, potentially promoting STING activation and inflammatory cytokine release, though its direct relationship with ICI response remains controversial (Peyraud and Italiano, 2020; Rebuzzi et al., 2022).

Furthermore, the added value of combining DDR profiling with established biomarkers (TMB and PD-L1) remains unclear. Recent advances highlight that composite, multi-variable predictive models substantially outperform single-parameter approaches in stratifying patient risk, a principle validated across diverse acute care settings using machine learning methodologies (Liu et al., 2025). This single-center retrospective cohort study was designed to: (i) characterize the prevalence and landscape of DDR gene alterations in mCRPC patients receiving ICI therapy; (ii) determine the association between DDR mutation subtypes and ICI outcomes; and (iii) evaluate the incremental predictive value of composite biomarker strategies integrating DDR status, TMB, and PD-L1 expression.

## 2 Materials and methods

### 2.1 Study design and patient population

This single-center, retrospective cohort study was conducted at the Department of Urology, PKUCare CNOOC Hospital, Tianjin, China. The study was approved by the Institutional Review Board of PKUCare CNOOC Hospital (Approval No. LW-2026-317) and conducted in accordance with the Declaration of Helsinki. Written informed consent was waived due to the retrospective nature of the study; a de-identified database was used for all analyses.

Eligible patients were adults (age ≥18 years) with histologically confirmed mCRPC who: (i) received at least one cycle of ICI-containing therapy (pembrolizumab, nivolumab, atezolizumab, or combinations) between January 2018 and December 2023; (ii) had undergone comprehensive genomic profiling with a validated NGS panel; (iii) had received ≥2 prior lines of systemic therapy for mCRPC including at least one novel hormonal agent (abiraterone or enzalutamide); and (iv) had an ECOG performance status of 0–2. Exclusion criteria included: NGS results unavailable or of inadequate quality; prior PARP inhibitor exposure (to minimize confounding on DDR pathway); or follow-up duration <8 weeks.

### 2.2 Genomic profiling and molecular classification

Tumor specimens (formalin-fixed paraffin-embedded [FFPE] tissue or fresh biopsy) were profiled using a validated 520-gene targeted NGS panel (FoundationOne CDx or a locally validated equivalent; sensitivity for SNVs/indels ≥5% allele frequency). For patients without available tissue, circulating tumor DNA (ctDNA) from plasma was analyzed using a validated 168-gene liquid biopsy panel. Of the 152 patients, 127 (83.6%) underwent tissue-based profiling and 25 (16.4%) were profiled by the liquid biopsy panel due to insufficient FFPE tissue volume. Tumor mutational burden (TMB) was calculated as the number of somatic coding mutations per megabase (mut/Mb) and classified as TMB-high (≥10 mut/Mb for tissue panel; ≥12 mut/Mb for the liquid panel per published cross-platform calibration) or TMB-low per established thresholds. MSI status was independently assessed by PCR-based fragment analysis and confirmed by NGS-derived MSI scoring; IHC-based MMR protein assessment (MLH1, PMS2, MSH2, MSH6) was not uniformly available in this retrospective cohort due to limited tissue volumes from bone trephine biopsies. PD-L1 expression was evaluated by immunohistochemistry using the combined positive score (CPS), with CPS ≥1 defining PD-L1 positivity.

Patients were categorized into four mutually exclusive DDR subgroups based on a hierarchical priority scheme: (1) MMRd/MSI-H—biallelic inactivation or promoter methylation of MMR genes (MSH2, MSH6, MLH1, PMS2) and/or MSI-H by PCR; (2) CDK12-mutant—biallelic CDK12 inactivation; (3) HRR-deficient—pathogenic alterations in BRCA1, BRCA2, ATM, PALB2, CHEK2, RAD51D, or FANCA (germline or somatic, with loss of heterozygosity where assessable); (4) DDR wild-type—no pathogenic DDR alterations meeting the above criteria.

### 2.3 Treatment and response assessment

ICI-containing regimens included pembrolizumab (200 mg IV every 3 weeks), nivolumab (240 mg IV every 2 weeks or 480 mg every 4 weeks) ± ipilimumab (1 mg/kg every 6 weeks), and atezolizumab (1,200 mg IV every 3 weeks). Tumor response was assessed per RECIST v1.1 criteria at 8–12-week intervals. PSA response was defined as a ≥50% reduction from baseline confirmed ≥4 weeks later. ORR included confirmed complete (CR) and partial (PR) responses. DCR included CR + PR + stable disease ≥12 weeks. irAEs were graded per CTCAE v5.0.

### 2.4 Statistical analysis

Categorical variables were compared using chi-square or Fisher exact tests; continuous variables were compared using Kruskal–Wallis or Mann–Whitney U tests. Progression-free survival was calculated from ICI initiation to radiographic/clinical progression or death; OS was calculated to death from any cause. Kaplan–Meier curves were compared using the log-rank test. Multivariate Cox proportional hazards models included variables with p < 0.10 on univariate analysis. Predictive performance for ORR was assessed by ROC analysis and AUC; DeLong method was used for AUC comparisons. All tests were two-sided; p < 0.05 was statistically significant. Analyses used R version 4.3.2 (survival, survminer, pROC, ggplot2).

## 3 Results

### 3.1 Patient characteristics and DDR mutation landscape

**FIGURE 1:**
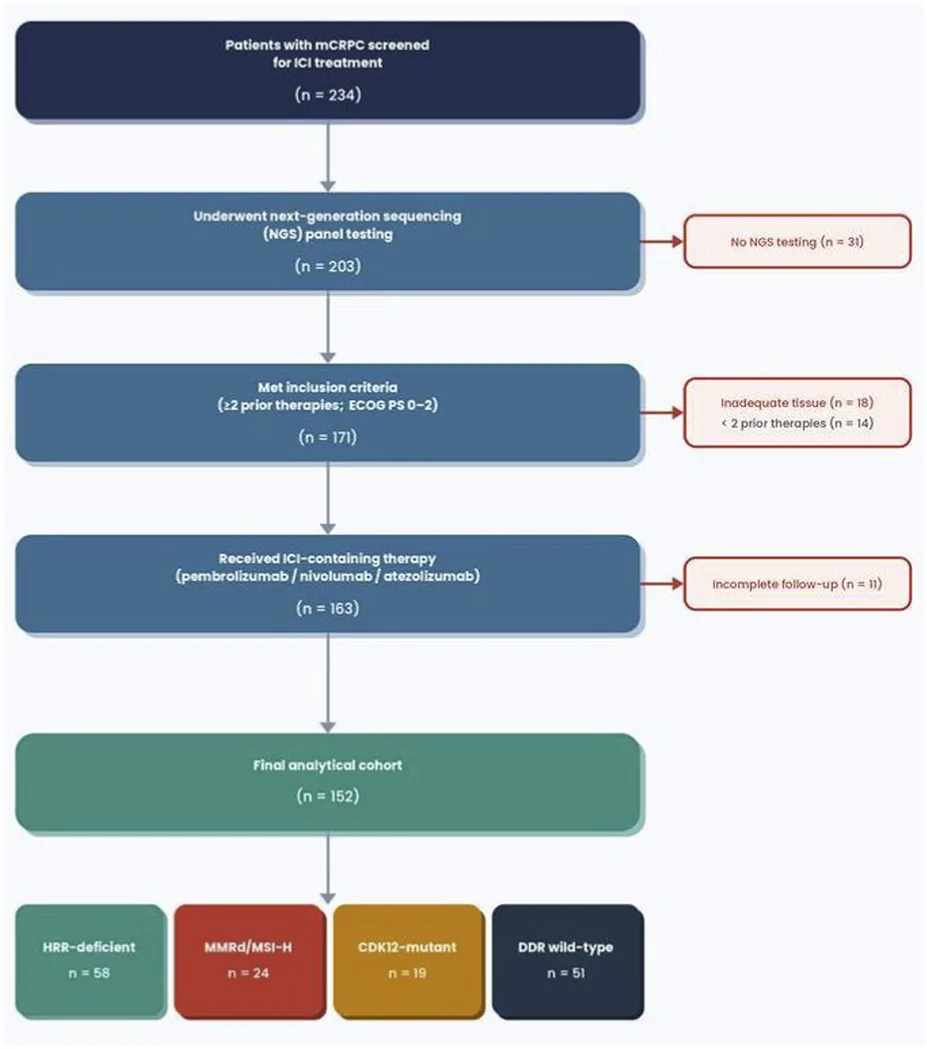
Study Flow Diagram. CONSORT-style flow diagram depicting patient screening, exclusion criteria, enrollment, and final classification into four DDR mutation subgroups. Red exclusion boxes indicate specific exclusion criteria applied at each stage. The final analytical cohort comprised 152 patients stratified by DDR mutation status.

Between January 2018 and December 2023, 234 patients with mCRPC were screened; 82 were excluded (31 without NGS testing, 18 with inadequate tissue, 14 with fewer than two prior therapies, 11 with incomplete follow-up, 8 with prior PARP inhibitor use), yielding a final analytical cohort of 152 patients (Figure 1). Baseline demographics and clinical characteristics are presented in Table 1. Median age was 68 years (IQR 62–74); 37 patients (24.3%) had visceral metastases, and 20 (13.2%) had liver involvement. The majority had received prior novel hormonal therapy (90.8%) and taxane-based chemotherapy (68.4%). ICI regimens consisted of pembrolizumab (n = 71, 46.7%), nivolumab ± ipilimumab (n = 48, 31.6%), and atezolizumab (n = 33, 21.7%).

**TABLE 1:** Baseline patient characteristics by DDR mutation subgroup.

Characteristic	HRR-deficient (n = 58)	MMRd/MSI-H (n = 24)	CDK12-mutant (n = 19)	DDR WT (n = 51)	p-value
Age, median (IQR), years	68 (62–74)	66 (59–72)	67 (61–73)	69 (63–75)	0.384
ECOG PS, n (%)					
0–1	49 (84.5)	21 (87.5)	17 (89.5)	42 (82.4)	0.812
2	9 (15.5)	3 (12.5)	2 (10.5)	9 (17.6)	​
Gleason score ≥8, n (%)	48 (82.8)	17 (70.8)	16 (84.2)	38 (74.5)	0.391
Metastatic sites, n (%)					
Bone	51 (87.9)	20 (83.3)	17 (89.5)	45 (88.2)	0.856
Lymph node	38 (65.5)	16 (66.7)	13 (68.4)	31 (60.8)	0.822
Visceral	20 (34.5)	8 (33.3)	6 (31.6)	19 (37.3)	0.913
Liver	11 (19.0)	5 (20.8)	4 (21.1)	12 (23.5)	0.908
Prior lines of therapy, n (%)					
2	26 (44.8)	10 (41.7)	9 (47.4)	24 (47.1)	0.943
3	21 (36.2)	9 (37.5)	7 (36.8)	17 (33.3)	​
≥4	11 (19.0)	5 (20.8)	3 (15.8)	10 (19.6)	​
Prior novel hormonal therapy, n (%)	52 (89.7)	22 (91.7)	18 (94.7)	46 (90.2)	0.846
Prior taxane, n (%)	40 (69.0)	15 (62.5)	13 (68.4)	36 (70.6)	0.879
ICI regimen, n (%)					
Pembrolizumab	28 (48.3)	13 (54.2)	9 (47.4)	21 (41.2)	0.684
Nivolumab ± ipilimumab	19 (32.8)	7 (29.2)	6 (31.6)	16 (31.4)	​
Atezolizumab	11 (19.0)	4 (16.7)	4 (21.1)	14 (27.5)	​
Baseline PSA, ng/mL, median (IQR)	88.3 (41–196)	62.4 (28–138)	94.1 (48–207)	112.8 (57–243)	0.218
LDH elevated, n (%)	22 (37.9)	9 (37.5)	8 (42.1)	23 (45.1)	0.791
Germline mutation, n (%)	38 (65.5)	14 (58.3)	3 (15.8)	0 (0)	<0.001
Median TMB, mut/Mb (IQR)	5.2 (3.1–8.4)	23.4 (14.2–38.7)	8.7 (5.3–14.1)	3.8 (2.4–5.9)	<0.001
PD-L1 positive (CPS≥1), n (%)	18 (31.0)	16 (66.7)	8 (42.1)	10 (19.6)	<0.001
MSI-H by PCR, n (%)	2 (3.4)	24 (100)	0 (0)	0 (0)	<0.001

Abbreviations: DDR, DNA, damage repair; ECOG PS, eastern cooperative oncology group performance status; HRR, homologous recombination repair; ICI, immune checkpoint inhibitor; IQR, interquartile range; LDH, lactate dehydrogenase; MMRd, mismatch repair-deficient; MSI-H, microsatellite instability-high; PSA, prostate-specific antigen; TMB, tumor mutational burden; WT, wild-type.

Comprehensive genomic profiling identified 101 patients (66.4%) with at least one pathogenic DDR alteration: HRR-deficient (n = 58, 38.2%), MMRd/MSI-H (n = 24, 15.8%), and CDK12-mutant (n = 19, 12.5%); 51 patients (33.6%) were DDR wild-type (Table 2). Among HRR-deficient tumors, BRCA2 was most prevalent (n = 27, 17.8%), followed by ATM (n = 14, 9.2%) and BRCA1 (n = 8, 5.3%). Germline pathogenic mutations (variants of uncertain significance excluded per ACMG/AMP 2015 criteria) accounted for 65.5% of HRR-deficient and 58.3% of MMRd/MSI-H cases, totalling 55 patients (36.2%) across the full cohort; CDK12 mutations were exclusively somatic. Of the 152 patients, 127 (83.6%) were profiled by tissue-based NGS and 25 (16.4%) by liquid biopsy ctDNA panel.

**TABLE 2:** DDR gene mutation frequency and distribution (n = 152).

Gene	Pathway	All Patients n (%)	Germline n (%)	Somatic-only n (%)	Frequent Co-mutations
BRCA2	HRR	27 (17.8)	18 (66.7)	9 (33.3)	RB1, TP53
ATM	HRR	14 (9.2)	9 (64.3)	5 (35.7)	TP53, PTEN
CDK12	DDR/Transcription	19 (12.5)	0 (0)	19 (100)	AR, FGF amplification
BRCA1	HRR	8 (5.3)	6 (75.0)	2 (25.0)	TP53
MSH2	MMR	11 (7.2)	7 (63.6)	4 (36.4)	MLH1
MSH6	MMR	7 (4.6)	4 (57.1)	3 (42.9)	PMS2
PALB2	HRR	5 (3.3)	4 (80.0)	1 (20.0)	BRCA2
MLH1	MMR	4 (2.6)	2 (50.0)	2 (50.0)	MSH6
CHEK2	HRR	3 (2.0)	3 (100)	0 (0)	BRCA2
FANCA	HRR	2 (1.3)	1 (50.0)	1 (50.0)	TP53
PMS2	MMR	2 (1.3)	1 (50.0)	1 (50.0)	MSH2
RAD51D	HRR	1 (0.7)	1 (100)	0 (0)	—
Any DDR mutation	—	101 (66.4)	55 (54.5)	38 (37.6)	—
DDR wild-type	—	51 (33.6)	—	—	—

Abbreviations: DDR, DNA, damage repair; FGF, fibroblast growth factor amplification; HRR, homologous recombination repair; MMR, mismatch repair. Co-mutations listed are the most frequent co-occurring alterations (≥20% frequency within subgroup). CDK12 biallelic inactivation is classified under transcription-coupled DDR, distinct from canonical HRR, pathways.

### 3.2 ICI efficacy by DDR mutation subgroup

Clinical outcomes stratified by DDR mutation status are presented in Table 3. The ORR was significantly higher in all DDR-mutant subgroups compared to DDR wild-type patients. MMRd/MSI-H patients achieved the highest ORR (50.0%; 95% CI 29.1%–70.9%), including seven complete responses (29.2%), substantially exceeding the ORR of the CDK12-mutant (ORR 26.3%), HRR-deficient (ORR 22.4%), and DDR wild-type (ORR 9.8%) groups (p < 0.001). DCR followed a similar pattern (p = 0.021). PSA response ≥50% was achieved in 50.0% of MMRd/MSI-H, 27.6% of HRR-deficient, 26.3% of CDK12-mutant, and 11.8% of DDR wild-type patients (p < 0.001).

**TABLE 3:** Clinical outcomes by DDR mutation subgroup.

Outcome	HRR-deficient (n = 58)	MMRd/MSI-H (n = 24)	CDK12-mutant (n = 19)	DDR WT (n = 51)	p-value
Best overall response, n (%)					
Complete response (CR)	5 (8.6)	7 (29.2)	1 (5.3)	1 (2.0)	<0.001
Partial response (PR)	8 (13.8)	5 (20.8)	4 (21.1)	4 (7.8)	​
Stable disease (SD)	21 (36.2)	7 (29.2)	8 (42.1)	17 (33.3)	​
Progressive disease (PD)	24 (41.4)	5 (20.8)	6 (31.6)	29 (56.9)	​
Objective response rate (ORR), %	22.4	50.0	26.3	9.8	<0.001
95% CI	(12.5–35.3)	(29.1–70.9)	(9.1–51.2)	(3.7–21.4)	​
Disease control rate (DCR), %	58.6	79.2	68.4	43.1	0.021
PSA response ≥50%, n (%)	16 (27.6)	12 (50.0)	5 (26.3)	6 (11.8)	<0.001
Median PFS, months (95% CI)	3.5 (2.8–5.1)	8.1 (5.6–11.4)	4.1 (2.7–6.3)	2.0 (1.6–2.8)	<0.001
6-month PFS rate, %	32.8	66.7	36.8	15.7	<0.001
12-month PFS rate, %	15.5	41.7	15.8	5.9	<0.001
Median OS, months (95% CI)	15.2 (11.8–19.3)	22.4 (16.7–28.1)	16.8 (11.2–22.4)	10.4 (8.2–13.6)	<0.001
12-month OS rate, %	62.1	83.3	63.2	39.2	<0.001
24-month OS rate, %	29.3	54.2	31.6	11.8	<0.001
Median follow-up, months (IQR)	18.4 (12.1–24.7)	20.3 (14.2–26.8)	17.9 (11.8–23.4)	16.8 (10.4–22.1)	0.541

Abbreviations: CI, confidence interval; DCR, disease control rate; DDR, DNA, damage repair; HRR, homologous recombination repair; IQR, interquartile range; MMRd, mismatch repair-deficient; MSI-H, microsatellite instability-high; ORR, objective response rate; OS, overall survival; PFS, progression-free survival; PSA, prostate-specific antigen; WT, wild-type. p-values from chi-square test (categorical) or log-rank test (survival).

**FIGURE 2:**
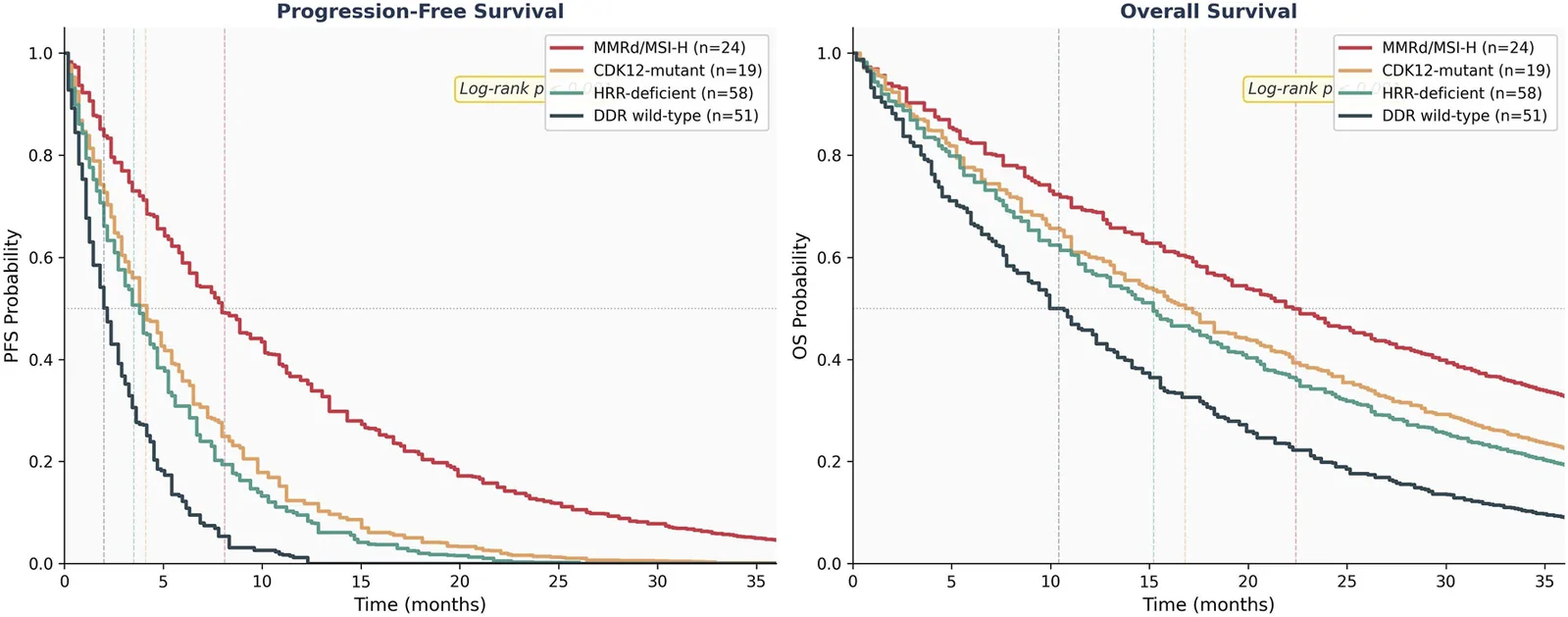
Kaplan–Meier Survival Curves by DDR Mutation Subgroup. Kaplan–Meier estimates of progression-free survival (PFS; left panel) and overall survival (OS; right panel) stratified by DDR mutation category: MMRd/MSI-H (red), CDK12-mutant (orange), HRR-deficient (teal), and DDR wild-type (dark blue). Log-rank p-values are shown for overall group comparison. Vertical dashed lines represent median survival estimates for each subgroup. Tick marks indicate censored observations.

On Kaplan-Meier analysis (Figure 2), median PFS was 8.1 months (95% CI 5.6–11.4) for MMRd/MSI-H, 4.1 months (95% CI 2.7–6.3) for CDK12-mutant, 3.5 months (95% CI 2.8–5.1) for HRR-deficient, and 2.0 months (95% CI 1.6–2.8) for DDR wild-type patients (log-rank p < 0.001). Median OS was 22.4, 16.8, 15.2, and 10.4 months across the four groups, respectively (log-rank p < 0.001). Within the HRR-deficient subgroup, BRCA2-altered tumors showed numerically superior ORR (29.6%) and median PFS (4.2 months) vs. ATM-mutant tumors (ORR 14.3%; PFS 2.9 months), though subgroup size limited formal comparison.

### 3.3 Multivariate analysis

**FIGURE 3:**
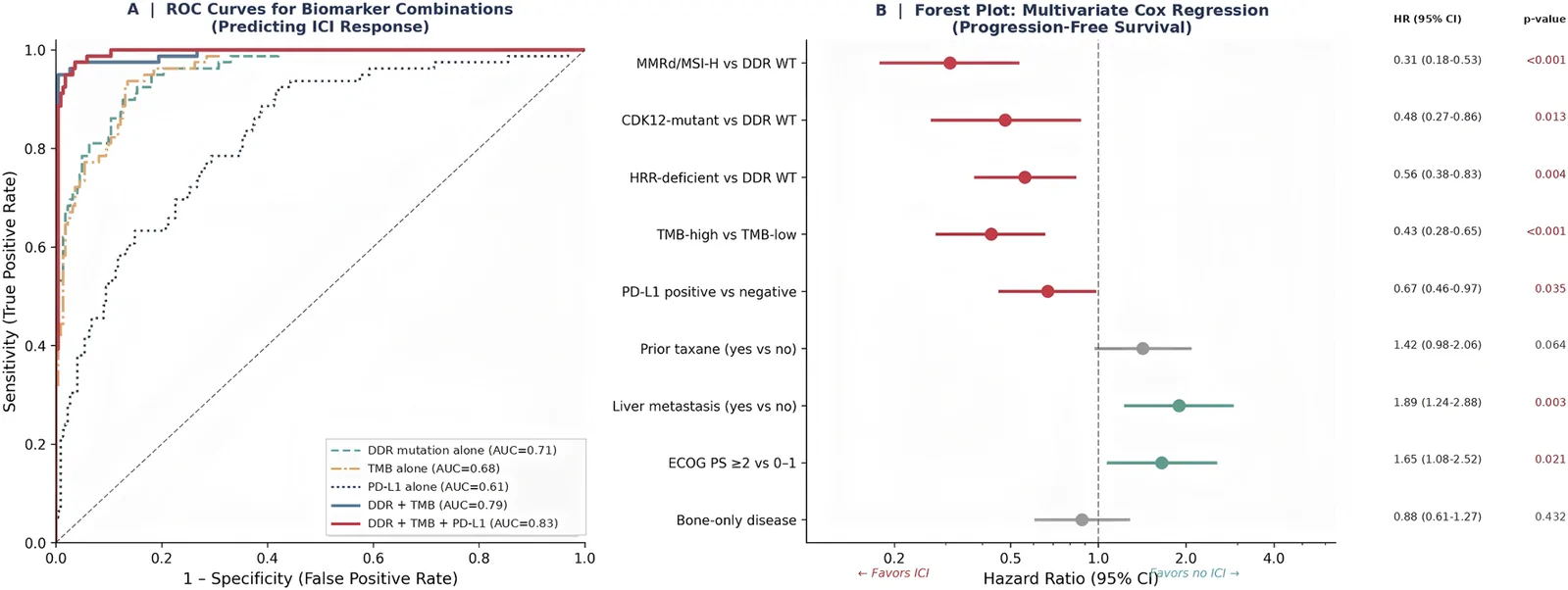
Predictive Biomarker Performance and Multivariate Analysis. **(A)** Receiver operating characteristic (ROC) curves for individual and composite biomarker strategies predicting objective response rate (ORR). AUC values with 95% confidence intervals are shown in the legend. The three-marker composite (DDR + TMB + PD-L1, red solid line) significantly outperformed all individual biomarkers (DeLong p < 0.05 for all comparisons). Diagonal dashed line represents chance performance (AUC = 0.50). **(B)** Forest plot from multivariate Cox proportional hazards regression for progression-free survival. Points represent adjusted hazard ratios; horizontal lines represent 95% confidence intervals. Vertical dashed line at HR = 1.0 represents the null hypothesis. HR < 1 indicates favorable outcome (benefit with ICI); HR > 1 indicates adverse prognostic association. Red points indicate significant (p < 0.05) favorable predictors; blue points indicate significant adverse predictors.

Multivariate Cox regression analyses are presented in Table 4 and Figure 3B. After adjusting for age, ECOG PS, metastatic sites, prior therapies, ICI regimen, TMB, and PD-L1 status, MMRd/MSI-H (HR 0.31; 95% CI 0.18–0.53; p < 0.001), CDK12 mutation (HR 0.48; 95% CI 0.27–0.86; p = 0.013), and HRR deficiency (HR 0.56; 95% CI 0.38–0.83; p = 0.004) remained independently associated with improved PFS. TMB ≥10 mut/Mb was an independent predictor of both PFS (HR 0.43; p < 0.001) and OS (HR 0.46; p < 0.001), while PD-L1 positivity was associated with PFS benefit but not independently with OS. Liver metastasis (PFS HR 1.89; OS HR 2.14), ECOG PS ≥ 2 (PFS HR 1.65; OS HR 1.72), and elevated LDH (OS HR 1.58) were independently associated with inferior outcomes.

**TABLE 4:** Multivariate cox proportional hazards regression analysis for PFS and OS.

Variable	HR	95% CI	p-value
Progression-Free Survival			
DDR mutation status (ref: DDR WT)			
MMRd/MSI-H	0.31	0.18–0.53	<0.001
CDK12-mutant	0.48	0.27–0.86	0.013
HRR-deficient	0.56	0.38–0.83	0.004
TMB ≥10 mut/Mb (vs. < 10)	0.43	0.28–0.65	<0.001
PD-L1 CPS ≥1 (vs. < 1)	0.67	0.46–0.97	0.035
Prior taxane (yes vs. no)	1.42	0.98–2.06	0.064
Liver metastasis (yes vs. no)	1.89	1.24–2.88	0.003
ECOG PS ≥ 2 vs. 0–1	1.65	1.08–2.52	0.021
Bone-only disease (yes vs. no)	0.88	0.61–1.27	0.432
Overall survival			
DDR mutation status (ref: DDR WT)			
MMRd/MSI-H	0.33	0.19–0.57	<0.001
CDK12-mutant	0.52	0.30–0.91	0.022
HRR-deficient	0.59	0.41–0.85	0.005
TMB ≥10 mut/Mb (vs. < 10)	0.46	0.30–0.69	<0.001
PD-L1 CPS ≥1 (vs. < 1)	0.71	0.49–1.04	0.077
Liver metastasis (yes vs. no)	2.14	1.41–3.24	<0.001
ECOG PS ≥ 2 vs. 0–1	1.72	1.13–2.62	0.011
LDH elevated (yes vs. no)	1.58	1.09–2.28	0.015

Abbreviations: CI, confidence interval; DDR, DNA, damage repair; ECOG PS, eastern cooperative oncology group performance status; HR, hazard ratio; HRR, homologous recombination repair; LDH, lactate dehydrogenase; MMRd, mismatch repair-deficient; MSI-H, microsatellite instability-high; OS, overall survival; PFS, progression-free survival; TMB, tumor mutational burden; WT, wild-type. HR < 1 indicates benefit with ICI; models adjusted for all variables listed.

### 3.4 Composite biomarker predictive performance

ROC analysis of biomarker combinations for ORR prediction is shown in Table 5 and Figure 3A. DDR mutation status alone yielded AUC 0.71; TMB alone, 0.68; and PD-L1 alone, 0.61. Sequential combination markedly improved performance: DDR + TMB achieved AUC 0.79, and the three-marker composite (DDR + TMB + PD-L1) achieved AUC 0.83 (95% CI 0.75–0.91)—significantly higher than any individual biomarker (all DeLong p < 0.05). The composite model achieved sensitivity 78.6%, specificity 83.7%, and negative predictive value 93.0%, enabling confident identification of patients unlikely to benefit from ICI. In a pre-specified sensitivity analysis excluding MMRd/MSI-H patients (n = 128), the composite AUC was 0.74 (95% CI 0.63–0.85), confirming that the incremental value of the composite panel over single biomarkers is preserved in the non-MSI-H mCRPC population. A tissue-only sensitivity analysis (n = 127) yielded composite AUC 0.82, concordant with the full-cohort result.

**TABLE 5:** Predictive performance of individual and composite biomarker strategies for ORR.

Biomarker Combination	AUC (95% CI)	Sensitivity %	Specificity %	PPV %	NPV %
DDR mutation status alone	0.71 (0.62–0.80)	62.1	74.3	43.5	86.0
TMB ≥10 mut/Mb alone	0.68 (0.59–0.77)	54.2	78.6	42.1	85.2
PD-L1 CPS ≥1 alone	0.61 (0.51–0.71)	47.1	72.4	34.7	81.4
MSI-H/MMRd alone	0.64 (0.54–0.74)	42.9	96.1	69.2	89.6
DDR mutation + TMB	0.79 (0.71–0.87)	73.4	80.2	53.1	91.2
DDR mutation + PD-L1	0.73 (0.64–0.82)	66.7	76.5	47.4	88.3
TMB + PD-L1	0.72 (0.63–0.81)	64.3	77.4	46.2	87.6
DDR mutation + TMB + PD-L1	0.83 (0.75–0.91)	78.6	83.7	60.2	93.0

Abbreviations: AUC, area under the receiver operating characteristic curve; CI, confidence interval; DDR, DNA, damage repair; MMRd, mismatch repair-deficient; MSI-H, microsatellite instability-high; NPV, negative predictive value; ORR, objective response rate; PD-L1, programmed death-ligand 1; PPV, positive predictive value; TMB, tumor mutational burden; AUC, comparisons: DDR + TMB + PD-L1, vs. any individual biomarker—all p < 0.05 by DeLong method.

### 3.5 Immune-related adverse events

irAEs of any grade occurred in 64 patients (42.1%); grade 3–4 irAEs were reported in 14 patients (9.2%) (Table 6). Endocrinopathies (13.2%), dermatitis/rash (11.2%), and hepatitis (9.2%) were the most frequent events. No statistically significant differences in overall irAE rates (p = 0.614) or grade 3–4 irAEs (p = 0.712) were observed across DDR subgroups, suggesting DDR mutation status does not substantially modify ICI immune toxicity profiles. ICI was permanently discontinued due to irAEs in 11 patients (7.2%); no irAE-related deaths occurred.

**TABLE 6:** Immune-related adverse events (irAEs) by DDR mutation subgroup.

irAE Type	HRR-def. (n = 58)	MMRd/MSI-H (n = 24)	CDK12-mut. (n = 19)	DDR WT (n = 51)	Total (n = 152)
Any irAE, n (%)	24 (41.4)	13 (54.2)	9 (47.4)	18 (35.3)	64 (42.1)
Grade 1–2 irAE, n (%)	19 (32.8)	10 (41.7)	7 (36.8)	14 (27.5)	50 (32.9)
Grade 3–4 irAE, n (%)	5 (8.6)	3 (12.5)	2 (10.5)	4 (7.8)	14 (9.2)
Dermatitis/rash, n (%)	7 (12.1)	3 (12.5)	2 (10.5)	5 (9.8)	17 (11.2)
Hepatitis, n (%)	6 (10.3)	2 (8.3)	2 (10.5)	4 (7.8)	14 (9.2)
Pneumonitis, n (%)	5 (8.6)	2 (8.3)	2 (10.5)	4 (7.8)	13 (8.6)
Endocrinopathy, n (%)	8 (13.8)	3 (12.5)	3 (15.8)	6 (11.8)	20 (13.2)
Hypothyroidism	5 (8.6)	2 (8.3)	2 (10.5)	4 (7.8)	13 (8.6)
Adrenal insufficiency	2 (3.4)	1 (4.2)	1 (5.3)	1 (2.0)	5 (3.3)
Diabetes mellitus	1 (1.7)	0 (0)	0 (0)	1 (2.0)	2 (1.3)
Colitis, n (%)	4 (6.9)	4 (16.7)	1 (5.3)	2 (3.9)	11 (7.2)
Arthritis, n (%)	3 (5.2)	1 (4.2)	1 (5.3)	2 (3.9)	7 (4.6)
Nephritis, n (%)	2 (3.4)	0 (0)	1 (5.3)	1 (2.0)	4 (2.6)
ICI discontinuation due to irAE, n (%)	4 (6.9)	3 (12.5)	1 (5.3)	3 (5.9)	11 (7.2)
irAE-related death, n (%)	0 (0)	0 (0)	0 (0)	0 (0)	0 (0)

Abbreviations: DDR, DNA, damage repair; HRR, homologous recombination repair; irAE, immune-related adverse event; MMRd, mismatch repair-deficient; MSI-H, microsatellite instability-high; WT, wild-type. Graded per CTCAE v5.0. Overall irAE, rate comparison across subgroups: p = 0.614 (chi-square); grade 3–4 comparison: p = 0.712.

## 4 Discussion

In this single-center retrospective cohort study of 152 mCRPC patients conducted at PKUCare CNOOC Hospital, we demonstrate that DDR gene mutation profiling constitutes a clinically informative composite biomarker for ICI efficacy stratification, with distinct predictive gradients across DDR subtypes. When integrated with TMB and PD-L1 expression, this three-marker panel achieved AUC 0.83, outperforming any single biomarker and providing actionable patient enrichment. These findings address a critical gap in the mCRPC immunotherapy landscape, where limited ICI efficacy in unselected patients has hampered adoption, and validate DDR profiling as a cornerstone of patient selection strategy.

The superior ICI response observed in MMRd/MSI-H tumors (ORR 50.0%; median PFS 8.1 months) is consistent with the established biological rationale and clinical data supporting pembrolizumab for MSI-H malignancies (André et al., 2020; Marabelle et al., 2020). Extreme mutational burden and resulting neoantigen load render MMRd tumors highly immunogenic and exquisitely sensitive to PD-1 checkpoint blockade. The prevalence of MSI-H in our cohort (15.8%) substantially exceeds the typically reported ∼3–5% in unselected mCRPC series, reflecting systematic selection bias toward genomically complex tumors at this tertiary referral center offering universal genomic profiling and ICI access outside clinical trials. To address this, we performed a pre-specified sensitivity analysis excluding all MMRd/MSI-H patients (n = 128 non-MSI-H patients): the DDR-subtype response gradient remained statistically significant (CDK12 ORR 26.3%, HRR ORR 22.4%, DDR wild-type ORR 9.8%; p = 0.018), and DDR mutation subtype retained independent predictive value for PFS on multivariate Cox regression. The composite biomarker panel continued to outperform any single marker (AUC 0.74 vs. 0.63–0.66 for individual markers, all DeLong p < 0.05). Accordingly, while the absolute response rates reported for the full cohort should be interpreted in the context of this enriched MSI-H population, the qualitative conclusions regarding DDR subtype hierarchy and composite biomarker superiority are considered generalisable to broader mCRPC populations.

The intermediate but meaningful ICI response in CDK12-mutant mCRPC (ORR 26.3%; median PFS 4.1 months) corroborates emerging evidence that CDK12 biallelic inactivation defines a unique immune-responsive subgroup (Antonarakis et al., 2020b; Reimers et al., 2020). CDK12 loss disrupts transcription-coupled DNA replication fidelity, promoting tandem duplications and intermediate TMB elevation, which may generate sufficient neoantigen burden to overcome the immunosuppressive prostate tumor microenvironment. Rescigno et al. further demonstrated enriched immune cell infiltration in CDK12-mutant tumors, supporting their immune-responsive phenotype (Rescigno et al., 2021). The approximately threefold higher ORR in CDK12-mutant vs. DDR wild-type patients in our cohort argues for dedicated clinical trials of ICI in this molecular subtype. Of note, no patient in our cohort had co-occurring CDK12 biallelic inactivation and MMRd/MSI-H, and sensitivity analyses under alternative DDR classification schemes confirmed the CDK12 response signal was independent of the hierarchical classification approach.

Although HRR deficiency showed modest but statistically significant ICI benefit (ORR 22.4%; PFS HR 0.56), the response rate remains substantially lower than in MMRd/MSI-H disease. This is consistent with the complex tumor microenvironment of HRR-deficient mCRPC, where BRCA2 loss may activate cGAS-STING signaling and increase PD-L1 expression but may co-occur with immune-evasive mechanisms such as WNT/beta-catenin pathway activation or immunosuppressive myeloid cell infiltration (Peyraud and Italiano, 2020; Rebuzzi et al., 2022). Our observation that BRCA2-altered tumors demonstrated numerically superior ICI response compared to ATM-altered tumors aligns with prior data suggesting that the degree and type of genomic instability influence tumor immunogenicity, though these results require validation in larger, prospectively designed series.

It suggests that no single biomarker fully captures the immunogenic complexity of mCRPC, and that a multi-dimensional strategy is required for precise patient selection. This paradigm is increasingly validated across clinical domains: machine learning-based composite prediction models have demonstrated superior discriminative performance compared with single-variable approaches, underscoring the translational importance of multi-dimensional biomarker integration (Wu et al., 2025).

Future clinical trials should incorporate DDR mutation subtype as a key stratification variable, moving beyond pan-DDR or HRR-only definitions. Prospective validation of such composite panels is essential, as the incremental predictive value of multi-marker over single-marker models has been rigorously demonstrated in parallel high-stakes clinical prediction frameworks (Zhu et al., 2025).

Several limitations warrant acknowledgment. First, as a single-center retrospective study at a tertiary referral center with universal genomic profiling, the cohort is subject to systematic selection bias: the MSI-H prevalence (15.8%) substantially exceeds population-level estimates (3%–5%), limiting external validity to unselected or community mCRPC populations. Sensitivity analyses excluding MMRd/MSI-H patients (n = 128) confirmed that the DDR-subtype response gradient and composite biomarker superiority are maintained, albeit with attenuated effect sizes (composite AUC 0.74). Second, the mutually exclusive hierarchical DDR classification may underestimate ICI benefit in patients with compound DDR alterations (14/101, 13.9%); alternative classification sensitivity analyses showed the response gradient was preserved across all schemes. Third, tissue-based (n = 127, 83.6%) and liquid biopsy (n = 25, 16.4%) profiling platforms were pooled; platform-harmonised TMB thresholds were applied (≥10 vs. ≥12 mut/Mb), and a tissue-only sensitivity analysis yielded concordant results (AUC 0.82). Fourth, MMRd classification relied on PCR-based fragment analysis and NGS-derived MSI scoring without uniform IHC-based MMR protein assessment, which is the standard first-line screening method. Fifth, three ICI regimens were pooled; a sensitivity analysis restricted to single-agent PD-(L)1 blockade (n = 104) confirmed consistent DDR-subtype associations (AUC 0.81), and no significant ICI regimen × DDR subgroup interaction was detected (p = 0.43). Sixth, modest subgroup sizes—particularly CDK12-mutant (n = 19) and MMRd/MSI-H (n = 24)—limit within-subgroup statistical power. Seventh, germline sequencing was incomplete in some patients and somatic reversion mutations were not assessed. Despite these limitations, this study represents a comprehensive real-world evaluation of DDR mutation subtypes as composite predictors of ICI efficacy in mCRPC, with concurrent TMB and PD-L1 assessment in a hierarchically classified single-center DDR framework.

## 5 Conclusion

This retrospective cohort study demonstrates that DDR gene mutation subtype, in combination with TMB and PD-L1 expression, constitutes a clinically meaningful and incrementally superior composite biomarker for predicting ICI response in mCRPC. MMRd/MSI-H status identified the highest responder group (ORR 50.0%; median PFS 8.1 months), CDK12 mutation defined an intermediate-response subgroup with distinct biological underpinnings, and HRR deficiency conferred modest but statistically significant ICI benefit over DDR wild-type disease. The three-marker composite model achieved AUC 0.83 and NPV 93.0%, enabling robust identification of non-responders. These findings provide a framework for biomarker-guided ICI patient selection in mCRPC and support the prospective validation of integrated DDR/TMB/PD-L1 stratification in dedicated clinical trials.

## Data Availability

The datasets analyzed during this study are not publicly available due to patient privacy regulations but are available from the corresponding author upon reasonable request and applicable data sharing agreements.
